# National Consensus Catalogue of Psychomotor Competencies for Brazilian Medical Graduates: a modified Delphi Study – Part II

**DOI:** 10.1590/1806-9282.20261142

**Published:** 2026-10-19

**Authors:** Adriana Gibo Padanosque, José Eduardo Lutaif Dolci, César Eduardo Fernandes, Antônio José Gonçalves, Lucas de Oliveira Sassi, Francisco Sandro Menezes-Rodrigues, José Mario Podanosque, Fernando Sabia Tallo

**Affiliations:** 1 Universidade Federal de São Paulo – São Paulo (SP), Brazil.; 2 Santa Casa de São Paulo, Faculdade de Ciências Médicas – São Paulo (SP), Brazil.; 3 Faculdade de Medicina do ABC – Santo André (SP), Brazil.

**Keywords:** Education, medical, undergraduate, Competency-based education, Clinical competence, Delphi technique, Psychomotor performance, Curriculum

## Abstract

**OBJECTIVE:**

To develop Part II of the National Consensus Catalogue of essential psychomotor competencies and establish the minimum expected competence levels for graduating medical students across 11 clinical and diagnostic support specialties in Brazil using a modified Delphi process.

**METHODS:**

This national consensus-development study used a two-phase, two-round modified Delphi process coordinated by the Brazilian Medical Association. Preliminary specialty-specific catalogues were developed between February and June 2025 by Scientific Committees from 11 specialty societies, which also proposed the minimum expected competence level for each psychomotor competency statement. Round 1 of the modified Delphi process was conducted from August to December 2025, and Round 2 was conducted from February to June 2026. Board-certified physicians evaluated each competency-competence level combination using a five-point Likert scale. Consensus was predefined as ≥75% agreement. Statements achieving <60% agreement were excluded after Round 1, whereas those achieving 60.0–74.9% agreement were revised and reassessed in Round 2. Additional competency statements proposed during Round 1 were evaluated in Round 2.

**RESULTS:**

A total of 667 physicians participated in Round 1, and 499 completed Round 2. The preliminary catalogues comprised 346 psychomotor competency statements; 240 (69.4%) achieved consensus and 106 (30.6%) were excluded. Participants proposed 38 additional statements, of which 36 (94.7%) achieved consensus in Round 2. The Part II catalogue comprised 276 specialty-specific psychomotor competency statements, corresponding to 229 unique psychomotor competencies after harmonization. Most competency statements were classified as perform independently (n=195), followed by perform under supervision (n=62) and master the technique (n=19).

**CONCLUSION:**

This modified Delphi study established Part II of the National Consensus Catalogue of essential psychomotor competencies and minimum expected competence levels for graduating medical students, encompassing 11 clinical and diagnostic support specialties in Brazil.

## INTRODUCTION

The primary goal of undergraduate medical education is to prepare graduates for safe, effective, and patient-centered care by acquiring essential psychomotor competencies before supervised clinical practice. These competencies support diagnostic, therapeutic, and emergency procedures. However, substantial variability persists among medical schools regarding which competencies should be taught, assessed, and expected at graduation, underscoring the need for national educational standards^1,2^.

Over the past two decades, competency-based medical education has transformed undergraduate training, while national frameworks have defined expected outcomes across educational systems^3,4^. Modified Delphi studies have also defined undergraduate competencies and Entrustable Professional Activities in specific contexts^5,6^. Despite these advances, most frameworks emphasize broad competency domains rather than essential psychomotor competencies and minimum expected competence levels at graduation. Consequently, no National Consensus Catalogue has comprehensively defined these competencies across major clinical and diagnostic support specialties.

In Brazil, the National Curriculum Guidelines define general competencies expected of graduating physicians but neither establish a standardized psychomotor competency catalogue nor specify minimum expected competence levels^7^. To address this gap, Scientific Committees from specialty societies affiliated with the Brazilian Medical Association (*Associação Médica Brasileira* – AMB) developed preliminary specialty-specific catalogues that subsequently underwent a structured modified Delphi process according to DELPHISTAR and ACCORD recommendations^8,9^.

The National Consensus Catalogue of Psychomotor Competencies for Brazilian Medical Graduates was developed as a national initiative to define essential psychomotor competencies and their minimum expected competence levels at graduation across multiple medical specialties. Part I comprised the initial set of specialty-specific catalogues developed within this national initiative using the same structured consensus approach. The present study represents Part II and extends the initiative to Cardiology, Dermatology, Gastroenterology, Gynecology and Obstetrics, Adult Intensive Care Medicine, Neurology, Ophthalmology, Orthopedics and Traumatology, Otorhinolaryngology, Pediatrics, and Pulmonology. The study aimed to develop consensus-based specialty-specific catalogues of essential psychomotor competencies and establish their minimum expected competence levels for graduating medical students across these specialties using a modified Delphi process.

## METHODS

### Study design

This national consensus-development study used a two-phase modified Delphi design to develop Part II of the National Consensus Catalogue of essential psychomotor competencies and establish minimum expected competence levels for graduating medical students across 11 clinical and diagnostic support specialties in Brazil. The study was coordinated by AMB and conducted according to the DELPHISTAR and ACCORD recommendations for consensus studies^8,9^. The modified Delphi method was chosen because of its established role in defining essential procedural competencies for undergraduate medical education^10^. The overall study design is presented in Figure 1.

**Figure 1:**
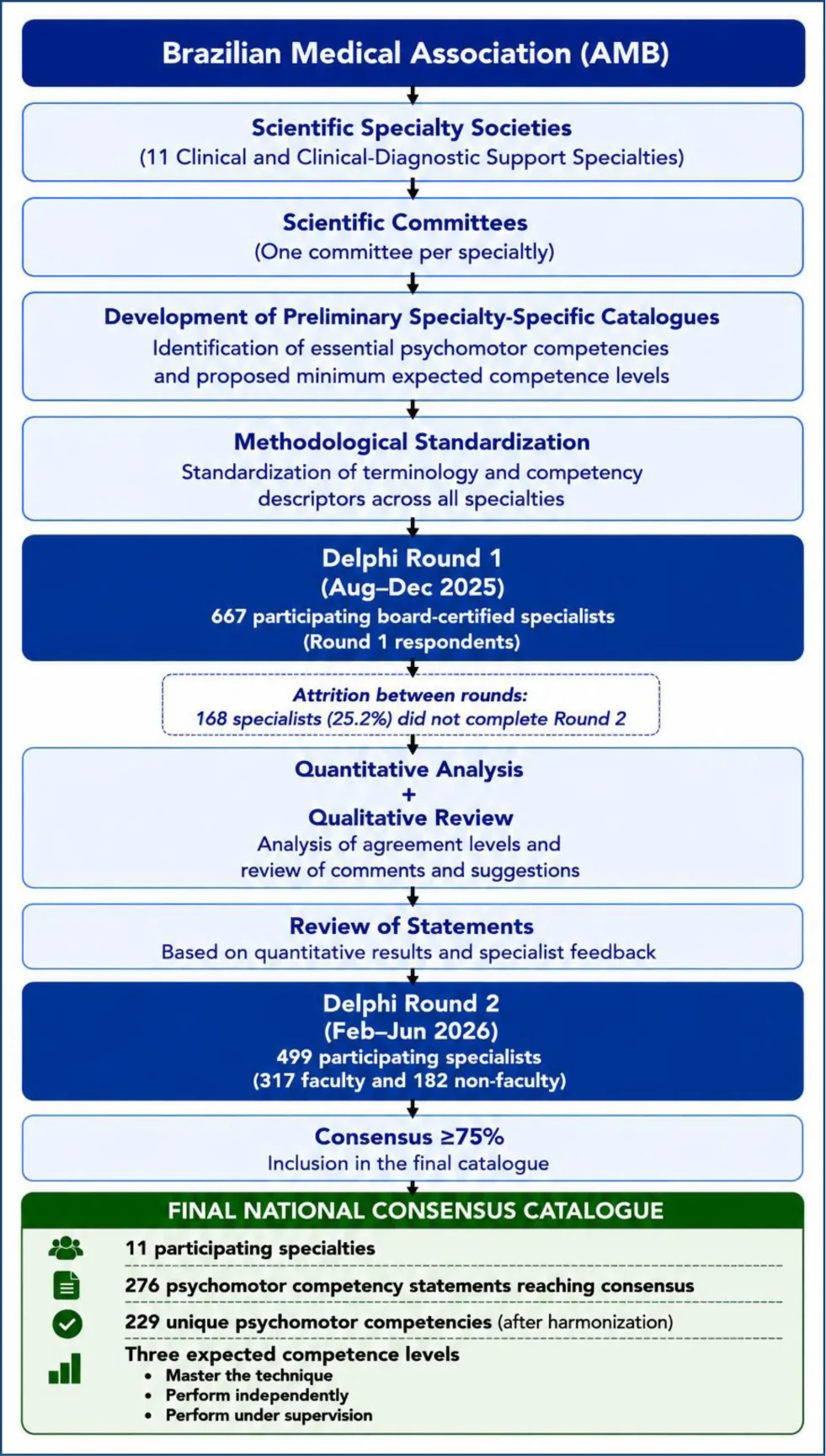
Modified Delphi process for development of a national consensus catalogue of psychomotor competencies.

### Phase 1: Development of preliminary catalogues

Phase 1 was conducted from February to June 2025. During this period, specialty societies affiliated with AMB were invited to participate in the national consensus initiative. Societies were included if they accepted the invitation and completed the development of their preliminary specialty-specific catalogue; societies that declined or did not complete this phase were not included. Participating societies appointed Scientific Committees, which independently developed the preliminary catalogues of psychomotor competencies for undergraduate medical education and proposed the minimum expected competence level for each competency: Master the technique, perform independently, or perform under supervision. The principal investigators subsequently standardized terminology, formatting, and wording while preserving the technical content of the preliminary catalogues^8,9^. Phase 1 did not involve recruitment of Delphi panelists or collection of Delphi questionnaire data. These procedures were conducted subsequently by the study investigators during Phase 2.

### Phase 2: Modified Delphi Consensus

The national modified Delphi process comprised two anonymous rounds conducted by the study investigators. Round 1 was conducted from August to December 2025. Potential panelists were identified from AMB national database of board-certified specialists. Physicians were eligible if they were board-certified and recognized by AMB in one of the specialties included in the study. Eligible specialists were invited individually by email to participate voluntarily, and the electronic Delphi questionnaire was distributed through the same communication channel. Round 2 was conducted from February to June 2026 among specialists who had completed Round 1.

Delphi panelists participated in an individual capacity and did not serve as official representatives of their respective specialty societies. Specialists who completed Round 1 but did not respond during Round 2 were considered lost to follow-up and were not replaced. No participant was excluded after enrollment for reasons other than nonresponse. The Delphi procedures followed contemporary recommendations for consensus studies^8,9^.

### Delphi questionnaire

Each questionnaire item presented one psychomotor competency and its proposed minimum expected competence level. Participants rated each competency-competence-level combination using a five-point Likert scale: strongly agree, partially agree, neither agree nor disagree, partially disagree, and strongly disagree.

When different competence levels were proposed for the same competency, each combination was evaluated independently. After Round 1, participants could suggest additional competencies, identify omissions, or recommend wording changes through an open-ended comment field. All qualitative suggestions were reviewed by the principal investigators, who assessed their relevance, clarity, and potential overlap with existing statements. Based on this review, suggestions were accepted, reformulated, merged with semantically similar statements, or excluded before inclusion in the Round 2 questionnaire^8,9^.

### Consensus criteria

Agreement was defined as the combined proportion of strongly agree and partially agree responses. Psychomotor competency statements achieving ≥75% agreement reached consensus. Statements with 60.0–74.9% agreement after Round 1 were revised and reassessed in Round 2, whereas those with <60% agreement were excluded. Additional statements retained after review of participant suggestions were included in the Round 2 questionnaire. Only statements achieving the predefined consensus threshold were incorporated into the National Consensus Catalogue^8,9^.

### Final catalogue and data analysis

The Delphi process evaluated 346 preliminary psychomotor competency statements across 11 specialties. After two rounds, the Part II catalogue comprised 276 specialty-specific psychomotor competency statements. Statements describing the same psychomotor procedure or clinical action across different specialties were considered semantically equivalent and were harmonized by the principal investigators. Disagreements were discussed among the principal investigators and resolved by majority decision. This process resulted in 229 unique psychomotor competencies in Part II. The overall study workflow is presented in Figure 1, and the distribution of psychomotor competency statements according to minimum expected competence level is shown in Table 1. Complete specialty-specific catalogues are provided in Supplementary Tables S16–S26. Subsequently, the unique psychomotor competencies derived from Parts I and II were consolidated into the Consolidated National Consensus Catalogue, comprising 319 unique psychomotor competencies organized into nine educational macrodomains (Supplementary Appendix 1). This organizational synthesis did not constitute an additional Delphi round.

**Table 1:** Distribution of final specialty-specific psychomotor competency statements by specialty, Round 2 Delphi panel size, and minimum expected competence level at medical graduation.

Specialty	Round 2 Delphi experts, n	Total competency statements, n	Master the technique, n	Perform independently, n	Perform under supervision, n
Cardiology	33	18	2	12	4
Dermatology	57	17	1	14	2
Gastroenterology	53	14	2	9	3
Gynecology and Obstetrics	50	28	1	25	2
Intensive Care Medicine	47	30	2	15	13
Neurology	45	25	2	20	3
Ophthalmology	43	25	1	18	6
Orthopedics and Traumatology	40	27	1	20	6
Otorhinolaryngology	35	22	1	18	3
Pediatrics	66	51	3	34	14
Pulmonology	30	19	3	10	6
Subtotal	499	276	19	195	62
Unique psychomotor competencies		229	—	—	—

The final National Consensus Catalogue comprised 276 specialty-specific psychomotor competency statements across 11 specialties, corresponding to 229 unique psychomotor competencies after harmonization. Minimum expected competence levels were classified as *Master the technique*, *Perform independently*, or *Perform under supervision*. The number of Delphi experts completing Round 2 in each specialty is also shown.

## RESULTS

### Characteristics of the expert panel

A total of 667 board-certified physicians participated in Round 1, and 499 completed Round 2, corresponding to an overall attrition rate of 25.2%. Specialty-specific participation, attrition, professional experience, academic appointment, and geographic distribution are summarized in Table 2. Among Round 2 participants, 317 (63.5%) held academic appointments and 182 (36.5%) were exclusively engaged in clinical practice. Participants represented all five Brazilian regions: Southeast (56.6%), South (19.4%), Northeast (11.2%), Central-West (7.9%), and North (4.9%).

**Table 2:** Characteristics of the Delphi study, expert panel, and specialty-specific participation across Delphi rounds.

Characteristic	Value
Participating specialties	11 specialties
Study design	National consensus-development study using a modified Delphi process
Delphi rounds	2
Round 1	August–December 2025
Round 2	February–June 2026
Consensus threshold	≥75% agreement
Items excluded after Round 1	<60% agreement
Items resubmitted to Round 2	60.0–74.9% agreement
Round 1 panel	667 participants
Round 2 panel	499 participants
Overall attrition	168/667 (25.2%)
Geographic distribution	Southeast 56.6%; South 19.4%; Northeast 11.2%; Central-West 7.9%; North 4.9%
Faculty members	317 (63.5%)
Non-faculty participants	182 (36.5%)
Professional experience in specialty	<5 years: 8.7%; 5–10 years: 18.7%; 10–15 years: 41.3%; >15 years: 31.3%
Specialty-specific panel composition	Round 1, n; Round 2, n; attrition, n (%)
Cardiology	58; 33; 25 (43.1)
Dermatology	65; 57; 8 (12.3)
Gastroenterology	84; 53; 31 (36.9)
Gynecology and Obstetrics	64; 50; 14 (21.9)
Adult Intensive Care Medicine	57; 47; 10 (17.5)
Neurology	65; 45; 20 (30.8)
Ophthalmology	50; 43; 7 (14.0)
Orthopedics and Traumatology	58; 40; 18 (31.0)
Otorhinolaryngology	47; 35; 12 (25.5)
Pediatrics	81; 66; 15 (18.5)
Pulmonology	38; 30; 8 (21.1)
Total	667; 499; 168 (25.2)
Final national catalogue	276 psychomotor competency statements reaching consensus
Unique psychomotor competencies	229

Data are presented as number (percentage) unless otherwise indicated. Specialty-specific panel composition is reported as the number of participants in Round 1, the number completing Round 2, and attrition between rounds. Attrition was calculated as the difference between the number of participants in Round 1 and Round 2 divided by the number of participants in Round 1, multiplied by 100. Professional experience refers to years of practice in the participant’s specialty.

### Delphi consensus process

The preliminary catalogues comprised 346 specialty-specific psychomotor competency statements. Following the first Delphi round, 240 (69.4%) reached consensus and 106 (30.6%) were excluded. Participants proposed 38 additional competency statements, of which 36 (94.7%) achieved consensus in the second round. The Part II catalogue comprised 276 specialty-specific psychomotor competency statements.

### Evolution of competency statements

The evolution of competency statements varied across specialties. Pediatrics developed the largest preliminary catalogue (60 statements), whereas Dermatology developed the smallest (8 statements). Dermatology showed the greatest proportional expansion following participant feedback, with 9 of 11 additional competency statements achieving consensus. Overall, 36 of the 38 newly proposed competency statements (94.7%) achieved consensus.

### Part II National Consensus Catalogue

After harmonization and removal of semantically equivalent competencies shared across specialties, the Part II catalogue comprised 229 unique psychomotor competencies derived from 276 specialty-specific psychomotor competency statements. Most competency statements were classified as perform independently (n=195), followed by perform under supervision (n=62) and master the technique (n=19) (Table 1). The complete specialty-specific catalogues are presented in Supplementary Tables S16–S26.

## DISCUSSION

This national modified Delphi study established the first Brazilian consensus catalogue of essential psychomotor competencies and minimum expected competence levels across 11 clinical and diagnostic support specialties. Together with Part I, it provides a national framework for competency-based undergraduate medical education. Competency-based medical education emphasizes explicit outcomes, progressive competence, and performance expectations^11,12^. National frameworks have supported curricular reform in Germany and the United Arab Emirates but generally address broad competency domains rather than specific psychomotor competencies and expected levels at graduation^2,3,4^. Curriculum mapping and milestone-based frameworks likewise support curriculum design without defining nationally agreed procedural expectations^13,14,15^. The present catalogue addresses this gap through national expert consensus. Most competency statements were classified as *Perform independently*, consistent with competency-based progression and entrustment, in which autonomy increases with demonstrated competence^16,17,18^. Contemporary educational models emphasize the level of supervision required for safe practice and preparation for independent professional activities^17,18,19^. Nationally agreed competence levels may therefore improve consistency in procedural training, assessment, and graduate performance across medical schools^20,21^.

An important contribution of this study was the integration of independently developed specialty-specific catalogues into a single national framework. Harmonization reduced 276 specialty-specific psychomotor competency statements to 229 unique psychomotor competencies, demonstrating substantial convergence among participating specialty societies. This common psychomotor core may facilitate curriculum mapping, reduce unnecessary duplication across disciplines, and promote integration of procedural teaching throughout undergraduate medical education^13,14,21^.

The modified Delphi process strengthened the catalogue by incorporating participant feedback. During Round 1, participants proposed 38 additional psychomotor competency statements, of which 36 achieved consensus in Round 2. Similar Delphi studies have identified essential procedural competencies for undergraduate medical education when empirical standards are unavailable, supporting this methodology for curriculum development^5,6,10^. Nevertheless, these findings provide evidence of content validity through expert consensus and should not be interpreted as psychometric validation or evidence of educational effectiveness.

The catalogue provides a practical framework for curriculum planning, simulation-based education, workplace learning, and competency-based assessment. Deliberate practice remains fundamental to psychomotor competence and requires repeated performance with structured feedback^22^. Simulation-based education also allows students to acquire and refine technical skills before clinical application, improving confidence and procedural performance in supervised settings^23,24^.

This study has several strengths. It was coordinated by AMB, involved Scientific Committees from 11 specialty societies, included a large panel of board-certified physicians, applied predefined consensus criteria, and followed DELPHISTAR and ACCORD recommendations^8,9^. Including faculty and non-faculty physicians from diverse practice settings broadened the range of perspectives represented in the consensus process. Previous research has demonstrated substantial diversity among Brazilian physicians, supporting broad professional representation in national medical education initiatives^25^.

### Limitations

Several limitations should be acknowledged. Participation was voluntary, introducing potential selection bias, and approximately one quarter of participants were lost between Delphi rounds. Although specialists from all five Brazilian regions participated, geographic representation was uneven, with a predominance of participants from the Southeast and limited representation from the North, which may restrict national representativeness. The findings reflect only the specialties that accepted the invitation and completed this project phase and should not be generalized to all specialties affiliated with AMB. Although the modified Delphi method provides evidence of content validity through expert consensus, it does not provide psychometric validation or evidence of educational effectiveness. Catalogue implementation, assessment properties, educational outcomes, and clinical impact were not evaluated and should be investigated in future studies across different educational and healthcare settings.

## CONCLUSIONS

This national modified Delphi study established Part II of the National Consensus Catalogue of essential psychomotor competencies and minimum expected competence levels for graduating medical students across 11 clinical and diagnostic support specialties in Brazil. The Part II catalogue comprised 276 specialty-specific psychomotor competency statements, corresponding to 229 unique psychomotor competencies after harmonization. Together with the competencies derived from Part I, these were subsequently integrated into the Consolidated National Consensus Catalogue, comprising 319 unique psychomotor competencies.

## Data Availability

The datasets generated and analyzed during the current study are available from the corresponding author upon reasonable request.

## References

[1] Matsuyama Y, Nomura O, Oikawa S, Kikukawa M, Shimizu I, Gomi H (2024). Competency-based medical education guidelines are context-based: lessons from national guidelines in five countries. Med Teach.

[2] Jennebach J, Özkaya J, Giesecke S, Meißner N, Seidel M, Wyszynski V (2026). National Competence-Based Catalogue of Learning Objectives for Undergraduate Medical Education (NKLM): process description over the last 15 years. GMS J Med Educ.

[3] Kar SS, Dube R, George BT, Bairy LK, Hajeer AH, Matalka IIA (2025). Implementation of United Arab Emirates competency framework for medical education in undergraduate medical curriculum. BMC Med Educ.

[4] Schumacher DJ, Kinnear B, Carraccio C, Holmboe ES, Busari JO, van der Vleuten CPM (2024). Competency-based medical education: the spark to ignite healthcare’s escape fire. Med Teach.

[5] Khan SJ, Yasmeen R, Imran S (2025). Building the 8-star doctor: a modified Delphi study to define, teach, and assess health professions education for undergraduate medical students. BMC Med Educ.

[6] Peters H, Holzhausen Y, Czeskleba A, Schumann M (2024). Defining a set of teaching EPAs tailored to an undergraduate medical curriculum using a modified Delphi approach. BMC Med Educ.

[7] Brasil (2014). Câmara de Educação Superior.

[8] Niederberger M, Schifano J, Deckert S, Hirt J, Homberg A, Köberich S (2024). Delphi studies in social and health sciences: recommendations for an interdisciplinary standardized reporting (DELPHISTAR). Results of a Delphi study. PLoS One.

[9] Gattrell WT, Logullo P, van Zuuren EJ, Price A, Hughes EL, Blazey P (2024). ACCORD (ACcurate Consensus Reporting Document): a reporting guideline for consensus methods in biomedicine developed via a modified Delphi. PLoS Med.

[10] Kodikara K, Seneviratne T, Premaratna R (2025). Identifying essential procedural skills for medical students: a modified Delphi technique. J Med Educ Curric Dev.

[11] Frank JR, Snell LS, Cate OT, Holmboe ES, Carraccio C, Swing SR (2010). Competency-based medical education: theory to practice. Med Teach.

[12] Englander R, Frank JR, Carraccio C, Sherbino J, Ross S, Snell L (2017). Toward a shared language for competency-based medical education. Med Teach.

[13] Murphy R, Barnes CJ, Enright PD, Gratton V, Bush SH (2024). Mapping an undergraduate medical education curriculum against national and international palliative care reference learning objectives. BMC Med Educ.

[14] Laird-Fick HS, DeMuth R, Gold J, Ferenchick G, Mavis B, Lipscomb W (2022). An exemplar milestone framework for scaffolding a competency-based medical school curriculum. Med Sci Educ.

[15] Boursicot K, Etheridge L, Setna Z, Sturrock A, Ker J, Smee S (2011). Performance in assessment: consensus statement and recommendations from the Ottawa conference. Med Teach.

[16] ten Cate O (2005). Entrustability of professional activities and competency-based training. Med Educ.

[17] Cate OT, Carraccio C, Damodaran A, Gofton W, Hamstra SJ, Hart DE (2021). Entrustment decision making: extending Miller’s pyramid. Acad Med.

[18] Sawatsky AP, O’Brien BC, Hafferty FW (2022). Autonomy and developing physicians: reimagining supervision using self-determination theory. Med Educ.

[19] Holmboe ES, Sherbino J, Englander R, Snell L, Frank JR, ICBME Collaborators (2017). A call to action: the controversy of and rationale for competency-based medical education. Med Teach.

[20] Gruppen LD, Mangrulkar RS, Kolars JC (2012). The promise of competency-based education in the health professions for improving global health. Hum Resour Health.

[21] Touchie C, Cate OT (2016). The promise, perils, problems and progress of competency-based medical education. Med Educ.

[22] Ericsson KA (2008). Deliberate practice and acquisition of expert performance: a general overview. Acad Emerg Med.

[23] Tallo FS, Vendrame LS, Baitello AL (2020). Comparison of three methods for teaching mechanical ventilation in an emergency setting to sixth-year medical students: a randomized trial. Rev Assoc Med Bras (1992).

[24] Elendu C, Amaechi DC, Okatta AU, Amaechi EC, Elendu TC, Ezeh CP (2024). The impact of simulation-based training in medical education: a review. Medicine (Baltimore).

[25] Tallo FS, Abib SCV, Baitello AL, Lopes RD (2014). An evaluation of the professional, social and demographic profile and quality of life of physicians working at the Prehospital Emergency Medical System (SAMU) in Brazil. Clinics (Sao Paulo).

